# Factors Related to Compliance with Recommendations for Hearing Aid Counseling: A Pilot Study

**DOI:** 10.3390/audiolres15050136

**Published:** 2025-10-11

**Authors:** Devora Brand, Cahtia Adelman, Dvora Gordon

**Affiliations:** 1 Speech and Hearing Clinic, Hadassah University Medical Center, Jerusalem 9112001, Israel; cahtiaa@hadassah.org.il (C.A.); dvorag@edu.jmc.ac.il (D.G.); 2 Department of Communication Disorders, Jerusalem Multidisciplinary College, Jerusalem 9101001, Israel

**Keywords:** hearing aids, counseling, compliance, barriers

## Abstract

**Objectives:** Hearing aids (HAs) are the most common intervention recommended for hearing loss (HL). Many adults with HL do not seek HA rehabilitation. Several studies have attempted to identify barriers and facilitators to using HAs. Different bureaucratic processes for acquiring HAs may lead to different barriers and facilitators. In addition, studies have not yet explored the factors influencing compliance with a recommendation for an HA consultation. This study focuses on the stage prior to consultation in a context where HAs are heavily subsidized. **Methods:** 148 patients who had undergone a hearing test during 2022 at Hadassah University Medical Center and received a recommendation to undergo a hearing aid consultation were contacted for a telephone survey. Seventy-two adults, 48 male and 24 female, aged 25–85 years, with HL ranging from slight to profound, responded to a telephone questionnaire. The questionnaire, based on two previously published English questionnaires and translated and adapted into Hebrew, was used to assess the main reasons a person did or did not comply with the recommendation to pursue an HA consultation. **Results:** HL was more severe in those who sought hearing rehabilitation. The main reasons for seeking hearing rehabilitation are the need and desire to hear better and pressure from others. The foremost reasons for not pursuing hearing rehabilitation are feeling that there is currently no need, esthetics, lack of time, and self-consciousness. No significant gender- or age-based differences were found. **Conclusions:** There are additional barriers to seeking HAs aside from cost and accessibility. Understanding the reasons for avoidance of hearing rehabilitation may help in developing strategies that encourage people to seek hearing rehabilitation and use HAs when the need exists.

## 1. Introduction

Acquired hearing loss in adults has been found to not only affect their ability to hear but is additionally correlated to cognitive decline [1], decreased quality of life [2], unemployment [3], and social isolation [4].

Hearing aids (HAs) are the most common intervention recommended for sensorineural hearing loss [5]. HA use has been found to improve speech perception and comprehension, thereby benefiting quality of life, general functioning, and interpersonal relationships [6,7,8]. Homan et al. [9] found that HA use decreased fatigue, listening effort, and negative feelings due to hearing loss. Many adults reported that they regretted not purchasing and using HAs earlier [10].

The World Health Organization [11] estimates that five percent of the world population has hearing loss that could benefit from a hearing device. Recent reviews underscore that this burden is increasing globally, emphasizing the need for efforts to be made by health workers and improved strategies [11,12]. However, despite the adverse effects of untreated hearing loss and the wide availability of hearing devices, many adults with hearing loss do not seek HAs or personal amplifiers. Donahue et al. [13] found that less than 20 percent of adults with hearing loss in the United States seek HA rehabilitation. Over the years, the percentage has increased to about 30% [14], though this remains much lower than desired.

Different studies have tried to identify the barriers and facilitators to seeking HAs. In Kochkin’s MarkeTrak survey [15], the barriers identified were not recognizing the severity of their HL, mistaken beliefs regarding the abilities of HAs, stigma of HA use for the elderly or disabled, esthetics, and being embarrassed that the HA will be visible. One major barrier reported was financial. A total of 64 percent of the participants stated financial reasons for not going ahead with HA fitting. Post-Foroosh et al. [16] and Knoetze et al. [17] found similar results. Jilla et al. [18] reported that financial concerns are a significant barrier to purchasing HAs in the USA. The primary reasons adults in the USA do not pursue HA uptake are concerns about their cost, difficulty adjusting to them, and the stigma associated with wearing hearing aids. Recent systematic reviews demonstrate that psychosocial barriers such as stigma, self-perception, and communication anxieties are significant deterrents to HA uptake despite advances in technology and accessibility of HAs [19].

### 1.1. Patient Demographics That Affect HA Use

For American adults, a relationship was found between family status, race, socio-economic status, and adopting an HA after recommendation. Married, Caucasian patients from high socioeconomic status were more likely to use an HA after receiving a recommendation to do so [20]. Meyer & Hickson [21] found a relationship between age and HA use for subjects 65 years old and older. The older the subject, the more likely they are to adopt HA use. Neither study found a difference between males and females [20,21].

### 1.2. Audiological Factors That Affect HA Use

Few studies examined the relationship between audiological factors, such as severity, shape, and type of hearing loss, and adult HA use. Lin et al. [22] calculated four-frequency pure tone averages (PTA) using 500 Hz, 1000 Hz, 2000 Hz, and 4000 Hz, and high-frequency averages of 3000 Hz, 4000 Hz, 6000 Hz, and 8000 Hz. They too found that the more severe the hearing loss, the more they used HAs.

Countries where the cost of HAs is significantly subsidized have higher usage rates [23], although market penetration remains under 50 percent. It would seem that other significant barriers to HA uptake must be involved. In Israel, HAs are both significantly subsidized and widely accessible. The Ministry of Health subsidizes about USD 1000 of the HA cost and obligates the health insurance companies to contract with HA importers for lower prices. This allows every citizen with a written recommendation from an audiologist and ENT doctor to acquire new HAs every 3.5 years with no co-payment or USD 100–1000 for advanced models, regardless of the degree of hearing loss. For reference, the average monthly salary in Israel is USD 4418.

**Figure 1:**
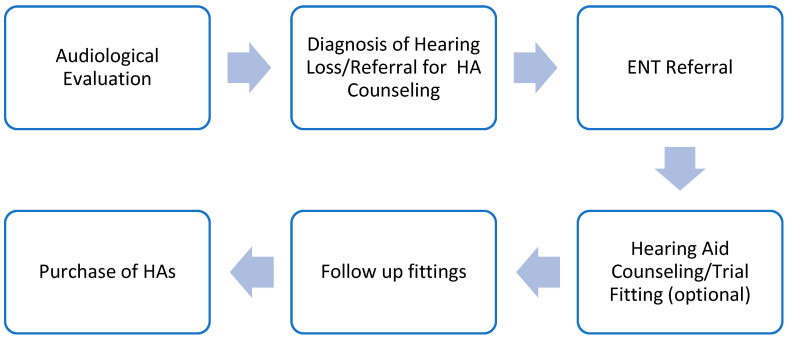
The pathway of hearing aid provision in Israel.

The insurance companies ensure there are accessible clinics throughout the country. The minimal costs involved for the end user in Israel provide an opportunity to examine the barriers and facilitators other than cost to pursuing the first step in the HA fitting process—the HA consultation. Figure 1 shows the pathway of hearing aid provision in Israel.

This study aims to identify the demographic, audiological, and attitudinal factors that influence compliance with recommendations for HA consultations among adults in Israel.

## 2. Materials and Methods

### 2.1. Participants

This study included 72 subjects between the ages of 26 and 85 years (M = 63.19, Sd = 14.28). All subjects underwent an audiological evaluation between 1 January 2022 and 1 October 2022 at the Hadassah University Medical Center. Hearing test results showed a range of sensorineural hearing loss from slight to profound, and all subjects received a recommendation to follow up with HA counseling.

Inclusion criteria were at least a slight (>15 dB) hearing loss in a four-frequency pure tone average (PTA): 500 Hz, 1000 Hz, 2000 Hz, and 4000 Hz [24,25]. Patients who had any previous experience with HAs were excluded.

Forty-eight males and 24 females participated. The participant ages ranged from 25 to 85 years and were categorized into two age groups: young adults (25–64 years), which included 19 males and 9 females, and older adults (65 to 85 years), comprising 29 males and 15 females. No differences were found between the distribution of gender in the two age groups (Chi sq (1) = 0.03, *p* = 0.54).

### 2.2. Research Tools

Questionnaire of seeking HA counseling—A phone questionnaire was developed, translated, and adapted for this study based on the English versions from Brooks & Hallam [24] and Saunders and Cienkowski [25]. The questionnaire consists of demographic information such as years of education and employment status and a subjective assessment of hearing and communication abilities. Additionally, it probed attitudes towards hearing loss and HAs and rated knowledge regarding the bureaucracy of purchasing HAs in Israel. Finally, subjects were asked to rate reasons for compliance/noncompliance with the recommendation to follow up with HA counseling (Supplemental Materials).

### 2.3. Demographic and Audiological Data

Audiograms were retrieved from the electronic hospital files, which included the audiological findings and recommendations as well. Demographic data of age and gender were also retrieved from these files.

### 2.4. Research Procedure

After receiving approval from the ethics boards of Hadassah University Medical Center and Hadassah Academic College, electronic files of patients were randomly chosen for patients 18 years and older who had a hearing test conducted between January and October 2022. A total of 148 files were found of patients with hearing loss who had received a recommendation for HA counseling. A total of 18 subjects did not answer the phone, 29 patients refused to participate in the study, 11 patients used/are using a HA, and 8 patients did not speak sufficient Hebrew or English to participate. A total of 72 patients gave consent to participate and completed the questionnaire verbally via telephone.

### 2.5. Statistical Analysis

Cramér’s V statistic was used to explore the relationships between demographic characteristics (e.g., age and gender), audiological characteristics (e.g., type, degree, and shape of hearing loss), and adherence to hearing aid counseling recommendations. To assess gender differences (males and females) in the primary reasons for following hearing aid counseling recommendations, an independent *t*-test for two samples was conducted. ANOVA was employed to evaluate potential differences between the two age groups concerning the reasons for following hearing aid counseling recommendations. Finally, Pearson correlation was used to examine the relationships between age and the various reasons for adhering to hearing aid counseling recommendations.

## 3. Results

### 3.1. Descriptives

Participants’ years of education ranged from 5 years to 25 years (M = 14.92, SD = 3.77), and their employment status was as follows: 30 were not working, and 42 were currently employed. Participants were not asked about their income.

#### 3.1.1. Demographic Factors

Between age and compliance with the recommendation for HA counseling (V = 0.20, *p* = 0.009, one-tail *p* < 0.05), a significant difference was found. The older group followed the recommendation for HA counseling more than the younger adult group, 40.91% vs. 21.43%. No association was found between gender and compliance with the recommendation (V = 0.06, *p* = 0.60). No association was found between employment status and compliance with the recommendation (V = 0.00, *p* = 1.00). Additionally, no significant difference in years of education was found between those who followed the recommendations (M = 15.33, SD = 4.26) and those who did not (M = 14.71, SD = 3.53) [t(70) = −0.66, *p* > 0.05].

#### 3.1.2. Audiological Factors

Degree of HL: No significant difference was found between those who followed the recommendations (M = 2.75, SD = 1.07) and those who did not (M = 2.33, SD = 1.03) regarding the severity of HL in the right ear [t(70) = −1.58, *p* > 0.05]. However, a significant difference was found for the severity of hearing loss in the left ear, with those who followed the hearing aid counseling recommendation showing more severe loss (M = 2.67, SD = 0.81) compared to those who did not follow the recommendation (M = 2.33, SD = 1.03) [t(70) = −1.58, *p* < 0.05]. Patients who sought HA counseling had more severe hearing loss.

No association was found between the type of HL (sensorineural, conductive, mixed) or the shape of the audiogram and adherence to the HA counseling recommendation (V = 0.21, *p* = 0.19; V = 0.24, *p* = 0.36). Similarly, no association was found between the symmetry of HL and following the recommendation for HA counseling (V = 0.06, *p* = 0.56).

### 3.2. Reasons for Compliance/Non-Compliance with the Recommendation

**Figure 2:**
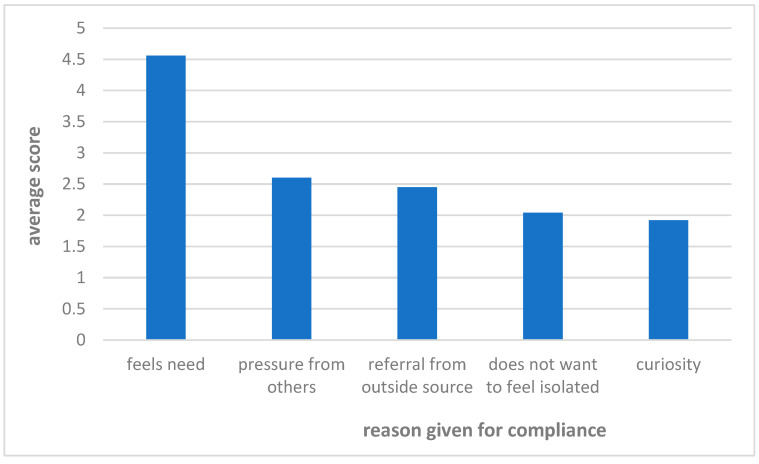
Reasons for compliance with HA counseling recommendation.

**Figure 3:**
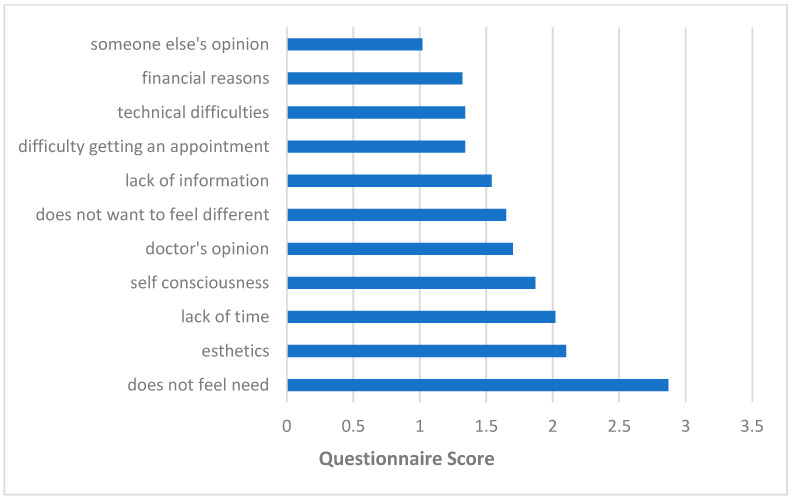
Reasons for non-compliance with HA counseling recommendation.

Out of the 72 participants, 24 sought HA counseling. The participants were asked to rate the reasons for their decision on a scale from 1 to 5. The primary reasons for compliance are shown in Figure 2 and were “the need to hear better” (M = 4.56, SD = 0.85), followed by “pressure from others” (M = 2.60, Sd = 1.76). The main reasons for non-compliance are shown in Figure 3 and were “did not feel a need” (M = 2.87, SD = 1.67), followed by “esthetics” (M = 2.20, SD = 1.54), “lack of time” (M = 2.02, SD = 1.43), and “self-consciousness” (M = 1.87, SD = 1.43).

No effect was found for age or gender regarding the reasons given for seeking or not seeking HA counseling.

No relationship was found between attitudes toward hearing loss and HA and compliance or noncompliance with HA counseling recommendations.

No difference was found between those who sought HA counseling (M = 2.08, SD = 1.31) and those who did not (M = 2.56, Sd = 1.58), t(69) = 0.21, *p* < 0.05, for the following findings. Similarly to the outcomes regarding the statement “a person with a hearing loss is perceived as old”, those who chose to comply (M = 1.91, SD = 1.20) and those who did not comply (M = 2.35, SD = 1.49) showed no significant difference [t(69) = 1.23, *p* < 0.05]. Similarly, no significant difference was found in the ratings of the statement “ a person with a HA is perceived as handicapped” [t(70) = −0.17, *p* < 0.05] or the statement “a HA can improve quality of life” [t(69) = −0.53, *p* < 0.05] between those who complied (M = 2.33, SD = 1.55; M = 4.29, SD = 1.12) and those that did not comply (M = 2.27, SD = 1.55; M = 4.14, SD = 1.02).

Regarding subjective assessment of hearing [t(70) = 1.58, *p* < 0.05] and communication abilities [t(70) = −1.81, *p* < 0.05], no differences were found between those that chose to seek HA counseling (M = 2.70, SD = 0.81; M = 3.29, SD = 0.90) and those who did not (M = 3.08, SD = 1.00; M = 2.81, SD = 1.12)

Knowledge of the bureaucracy involved in acquiring an HA in Israel did not seem to impact following up with HA counseling (M = 3.91, SD = 1.41) or not (M = 3.75, SD = 1.49) [t(70) = −0.45, *p* < 0.05].

## 4. Discussion

The aim of this study was to examine the barriers and facilitators to compliance with recommendations for HA consultation in the context of subsidized hearing aids. The relationship between demographic factors, such as gender and age, and audiological factors, such as the degree, shape, and type of hearing loss, was examined in relation to whether individuals sought or did not seek hearing rehabilitation after receiving a recommendation.

The findings of this study reveal a significant relationship between age and the decision to seek or not seek hearing rehabilitation after a recommendation. Gender, however, was not found to affect seeking HA counseling. These results are consistent with the Simpson et al. [20] study, which also found that gender did not influence the acquisition of HAs following a recommendation. They are also in line with Meyer & Hickson [21], finding a relationship between patients’ age and their willingness to seek hearing rehabilitation, with younger patients being less likely to pursue it.

Additionally, the findings revealed no differences in employment status (employed/unemployed) between those who sought rehabilitation and those who did not. This differs from Kochkin’s [26] study, which identified a relationship between employment status and HA use, with a higher percentage of employed individuals seeking rehabilitation. The current study had a high proportion of participants over 65 years of age who are retired, compared to a sample with more participants under 65 who are still employed. Furthermore, for those who reported being employed, their jobs may not require significant communication, meaning that hearing loss has less impact on their professional functioning and, therefore, may not lead to a strong need for rehabilitation. Another factor could be the subsidization of HAs by the government in Israel, which reduces the economic barrier to seeking rehabilitation, allowing unemployed individuals to afford HA consultation and purchase, which can be performed at no cost for the individual. Future studies could benefit from including a wider age range to further explore these factors.

Regarding audiological factors such as the severity of hearing loss, significant differences were found between those who sought rehabilitation and those who did not. Specifically, the severity of hearing loss in the left ear was greater among those who sought rehabilitation compared to those who did not. This finding is similar to that of Lin et al. [22], who found that the more severe the hearing loss, the more likely participants were to seek rehabilitation through hearing aids. However, when examining the differences in hearing loss in the right ear, no significant differences were found. A possible explanation for this is the discrepancy in the average severity of hearing loss between the two ears in the overall sample. Specifically, 15 participants had asymmetric hearing loss, with more than half experiencing worse hearing in the right ear.

The main reasons for seeking hearing rehabilitation, according to the findings of this study, are the desire and need to hear better. Conversely, the main reason for not seeking rehabilitation after a recommendation to do so is the perception that it is unnecessary. This suggests that individuals’ subjective perceptions of their hearing loss impact their decision to seek or not seek rehabilitation. Similar findings were noted by Kochkin [15] and Poost-Foroosh et al. [16], who found that the main reasons for avoiding hearing rehabilitation in the U.S. were attitudes toward hearing loss. However, we did not find significant differences in subjective ratings between the participants who sought and those who did not seek rehabilitation. More research is needed on the relationship between seeking HA consultation, acquiring HAs, HA satisfaction, and subjective hearing functionality.

Another key reason for seeking hearing rehabilitation identified in the current study is pressure from others, such as family members, to improve communication. This finding is supported by existing research, which shows that hearing rehabilitation through hearing aids can improve personal relationships [6,8].

The main barriers identified in this study were esthetics, failure to recognize a hearing loss, embarrassment about wearing hearing aids, and lack of time. These were reported similarly by both genders and across age groups. Understanding the rationale behind avoiding rehabilitation helps predict the challenges that may affect patients’ willingness to seek rehabilitation. These patients will need tailored solutions for the specific barriers they face, provided by doctors and audiologists. Raising awareness and offering proper guidance about the importance of hearing rehabilitation and the consequences of untreated hearing loss can lead to improved quality of life and relationships for these individuals. Moreover, understanding the reasons for not seeking rehabilitation can help shape a national strategy that encourages hearing rehabilitation by highlighting the negative consequences of untreated hearing loss on cognition and mental health, as well as improving access to the process and marketing hearing aids as more attractive. Since it seems that many who have undergone hearing testing will not comply with the recommendation for HA consultation, it may be beneficial to reassess the time and manner in which hearing test results are given. More time to discuss results and implications and HA options could facilitate greater compliance towards making an appointment for HA counseling. Another potential concern is the physical separation between clinics for audiological evaluations and HA consultation. Combining hearing tests with hearing aid consultations would make counseling more accessible.

It is important to note the significant limitations of our study. As a pilot study, we had a small sample; not many patients were keen or able to participate. It was important to contact them after enough time had lapsed so they could follow up with HA counseling if interested. It may be that some people need more time. An additional follow-up could be helpful. A larger group of participants with more representation of different ages would allow further understanding of different barriers and facilitators unique to different age groups.

The barriers and facilitators in other regions should be explored since cultural factors such as help-seeking and handicap stigma may be at play as well.

## 5. Conclusions

Financial barriers are often cited as primary obstacles to HA adoption. However, our study reveals that even in a highly subsidized health system, perceptual and psychosocial factors remain significant. Identifying barriers and motivators at the earliest stage of HA rehabilitation enables clinicians to address these barriers and improve not only individual patient outcomes but also reduce the broader societal impact of untreated hearing loss.

## Data Availability

The raw data supporting the conclusions of this article will be made available by the authors on request.

## Supplementary Materials

The following supporting information can be downloaded at: https://www.mdpi.com/article/10.3390/audiolres15050136/s1, File S1: Telephone Survey Questionnaire.
