# Supplementary material for: Factors Related to Compliance with Recommendations for Hearing Aid Counseling: A Pilot Study
Source: Audiol Res. 2025 Oct 11;15(5):136. doi: 10.3390/audiolres15050136 (PMC12561794; doi:10.3390/audiolres15050136)
Supplement: Supplementary file 1 [file audiolres-15-00136-s001.zip › audiolres-3778142-supplementary.pdf]

Supplementary materials: Telephone Survey Questionnaire

Pre-Questionnaire Opening (to be administered after obtaining informed consent)

- Did you use a hearing aid before performing a hearing test?
  ☐ Yes ☐ No

Telephone Questionnaire: Referral for Hearing Aid Consultation

1. How many years of education do you have?
2. Are you currently employed?
   ☐ Yes ☐ No
   - If yes or volunteer: What do you do? ________________
3. Did you return to a doctor after your hearing test?
   ☐ Yes ☐ No
   - If yes, which doctor?

Family doctor: ☐ Yes ☐ No Ear, Nose, Throat specialist: ☐ Yes ☐ No Other (specify): ________________

4. Do you remember what the doctor said regarding the recommendation for a hearing aid?
   ☐ Yes ☐ No
5. How would you rate the professional knowledge of the person who referred you for a hearing aid evaluation?
   (Scale: 1 = Not knowledgeable, 5 = Very knowledgeable)
   1 ☐ 2 ☐ 3 ☐ 4 ☐ 5 ☐
6. Did you seek a consultation for hearing aid fitting?
   ☐ Yes ☐ No
   - If you did NOT seek hearing aid consultation:
     - Why not?
       (Please rate each reason: 1 = Not at all, 5 = To a great extent)
     - Reason 1: ____________ 1 ☐ 2 ☐ 3 ☐ 4 ☐ 5 ☐
     - Reason 2: ____________ 1 ☐ 2 ☐ 3 ☐ 4 ☐ 5 ☐
     - (Add additional reasons as relevant)
   - If you DID seek hearing aid consultation:
     - Why did you go?
       (Please rate each reason: 1 = Not at all, 5 = To a great extent)
     - Reason 1: ____________ 1 ☐ 2 ☐ 3 ☐ 4 ☐ 5 ☐
     - Reason 2: ____________ 1 ☐ 2 ☐ 3 ☐ 4 ☐ 5 ☐
     - (Add additional reasons as relevant)

Self-Evaluation Scales

(For each, please rate from 1–5: 1 = Not at all, 5 = Very much so)

Question	1	2	3	4	5
How do you rate your hearing abilities?					
Do you experience communication difficulties in your daily life?					
In your opinion, can a hearing aid improve quality of life?					

Question	1	2	3	4	5
Do you perceive a person with a hearing aid as elderly?					
Do you perceive a person with a hearing aid as having a disability?					
Do you perceive a person with hearing loss as elderly?					
Do you perceive a person with hearing loss as having a disability?					
To what extent is the rehabilitation process towards hearing consultation clear to you?					
